# A cost-effective approach to measurements of fluorophore temperature sensitivity and temperature change with reasonable accuracy

**DOI:** 10.1038/s41598-024-57387-2

**Published:** 2024-03-21

**Authors:** Matthew Cai, Alexander Sun, Andrea Yan, Zachary Ding, Melvin Zunyao Jiang, Charissa Wang, Baohong Yuan

**Affiliations:** SRCP, RCLabX LLC, Southlake, TX 76092 USA

**Keywords:** Imaging and sensing, Characterization and analytical techniques

## Abstract

The demand for measuring fluorophore temperature sensitivity and temperature change in chemical or biological samples has spurred the search for effective methods. While infrared (IR) light-based thermal devices are popular, they are limited to surface temperature measurement. Fluorescence-based thermometry, which utilizes intensity, lifetime, polarization, and spectrum change, provides the temperature information directly from the samples and can have high temporal and spatial resolution. However, measuring fluorescence can be tricky and expensive. A cost-effective approach to achieving reasonable accuracy is highly desired. This study introduces such an approach, employing a light-emitting diode (LED) for fluorophore excitation and a laser diode (LD) for sample heating, with a phone camera recording fluorescence changes. A data processing method converts the video into digital data, processed through digital filters. Utilizing a small-volume cuvette enhances heating efficiency. This study serves as a practical guide for inexperienced individuals, including students, instructors, and researchers, facilitating entry into the field and navigating the complexities of fluorescence-based thermometry.

## Introduction

Noncontact measurement of temperature change in chemical or biological samples has been widely needed in various applications^1–8^. One popular method for this purpose uses an infrared (IR) light-based thermal device or camera. However, this method is limited to only measuring the surface temperature of the sample and may not exactly represent the temperature change in the sample. This may be an issue when the sample must be positioned in a container (such as a cuvette) because the IR light-based device may measure the temperature of the container’s surface, rather than the sample temperature change. Another challenge that IR thermometry technology faces is low spatial resolution. This can be a significant issue when the sample is constrained in a very small space (such as in a single micro- or nano-pore or a small droplet) or a high spatial resolution thermal image is needed^9^.

Fluorescence-based thermometry as a noncontact measurement method has been intensively studied during the past decades^1–4,9,10^. Various methods have been developed by using fluorescence intensity, lifetime, polarization, and spectrum change to measure temperature change^1–4,9^. These technologies can also be implemented using a fluorescence microscope for achieving high temporal and spatial resolution of temperature measurement, which has shown broad applications when IR technology is not an option^11,12^. While further advancements in sensitivity, accuracy, stability, and other aspects of fluorescence-based thermometry (such as biocompatibility of fluorophores for biomedical applications) are needed^3,10,11,13^, developing a cost-effective system and method for a laboratory with a limited budget is highly desired for helping inexperienced researchers and students understand the mechanism of this technology and acquire hands-on skills in instrumentation and data processing. The beauty of such a system and method is that it involves several fundamental fields, such as optical, thermal, electronics, mechanical, coding, and data processing. Going through a research project using fluorescence-based thermometry can dramatically enhance researchers and students experience with a limited budget. With this motivation in mind, we selected to measure the relative change in fluorescence intensity from a single type of fluorophore to measure the temperature change and fluorophore temperature sensitivity because fluorescence intensity is much easier to measure compared with fluorescence lifetime, polarization, and spectrum responses^1,3,4^. Also, the relative change in fluorescence intensity from a single fluorophore can avoid not only the effect of fluorophore concentration but also the complexity of using multiple fluorophores^1,3^. However, detecting fluorescence intensity and its changes can be very tricky for people with limited experience because of various background noises (from excitation light, background fluorescence, and electronic noise). Achieving an acceptable signal-to-noise ratio based on the fluorescence intensity change may be the biggest hurdle for many students, instructors, and researchers. In this study, we provide detailed steps and explanations in both instruments and data processing methods so that readers can follow this protocol to acquire meaningful results, get familiar with the fluorescence technology, and further develop their systems and methods.

## Instruments and methods

### Fluorescence optical systems and samples

**Figure 1:**
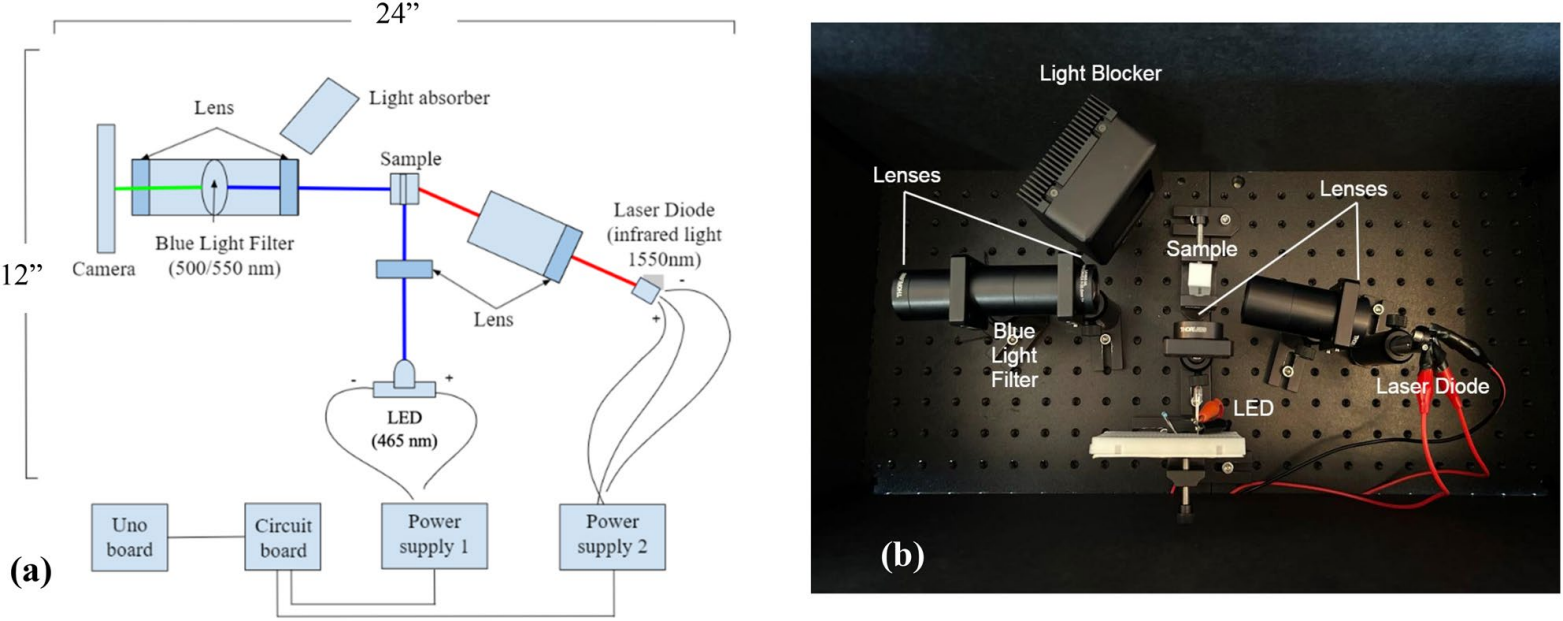
(**a**) A schematic diagram of the system (including optical, electronic and control systems); (**b**) a white light photo of the optical system.

**Figure 2:**
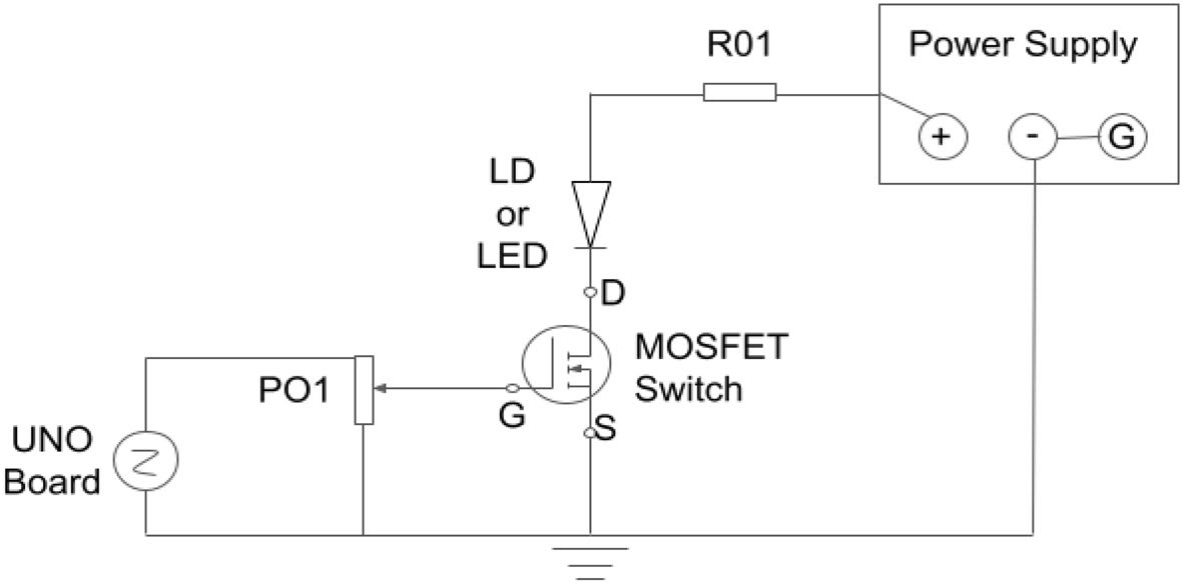
The schematic diagram of the circuit used to allow the UNO board to control the laser diode (LD) and light-emitting diode (LED). PO1: a voltage divider resistor; MOSFET: metal oxide semiconductor field-effect transmitter; UNO Board; microcontroller board based on the ATmega328P; D: drain; G: gate, controls the MOSFET; S: sink; R01: a resistor to limit the current for protection of LD and LED.

The schematic diagram of the fluorescence optical system is shown in Fig. 1a, and Fig. 1b is a white light photo of the system. The optical system includes two sub-systems: the fluorescence system and the heating system. Each system is powered by a power supply (KD3005D, Digikey, MN, USA) and controlled via a switching circuit as shown in Fig. 2.

The fluorescence system excites the sample via a light-emitting diode (LED) (LED465E, Thorlabs, NJ, USA) with a center wavelength of 465 ± 10 nm and a full-width-at-half-maximum (FWHM) of 25 nm. The voltage and current applied to the LED in this study are 2.7 V and 9 mA, respectively. The emitted blue light is focused on the sample in the cuvette via a lens (LA1805-ML, Thorlabs, NJ, USA). The fluorophore molecules of fluorescein 27 in the aqueous solution adopted in this study absorb the light and emit fluorescent light (it is also called fluorescein 548, C_20_H_10_O_5_Cl_2_, MW 401.20, a low-cost, water-soluble and non-toxic dye with a high quantum yield > 0.9 and a short lifetime 4 ns^1,14^). The emitted light is collimated by two lenses (LA1805-ML, Thorlabs, NJ, USA) and then focused on a mobile phone camera (iPhone 14 Pro, Apple, CA, US). In between the lenses, there are two long-pass emission filters—one that allows light with a wavelength longer than 500 nm to pass (FELH0500, Thorlabs, NJ, USA), and another that allows light with a wavelength longer than 550 nm to pass (FELH0550, Thorlabs, NJ, USA). By using these two emission filters, the background leakage of the excitation light from the LED can be minimized significantly more than by using one filter, which can help to reduce the background noise. This is mainly because the adopted filters are interference filters, which require light to be perpendicularly incident to the filters to achieve the nominal performance. In practice, this requirement may not be achieved perfectly because the excitation light can be scattered. This phenomenon has been well discussed in the literature^15,16^. The camera is manually controlled (to switch on or off) to record videos of the fluorescence variations during the experiments. The recorded videos are converted into digital data using our processing algorithms (see the “Data processing methods” section for details) and displayed in the figures. Note that the videos must be converted into data and processed by using digital filters to be able to visualize the fluorescence variations when temperature changes, which cannot be directly seen from the videos.

The fluorescence intensity of the adopted fluorophore (fluorescein 27^1^) has a positive correlation with temperature. Therefore, we expect that the fluorescence intensity will increase when the temperature rises and decrease when the temperature falls. To control the sample temperature, we also designed an optical heating system that serves to heat the sample in a cuvette (CV10Q7F, 0.7 ml micro fluorescence cuvette, Thorlabs, NJ, USA) using a high-power infrared (IR) laser diode (LD) (L1550G1, Thorlabs, NJ, USA). Its central wavelength is 1550 nm and it can be absorbed by water molecules to induce the temperature rise in the sample. The laser diode is powered by a power supply (KD3005D, Digikey, MN, USA). The voltage and current applied to the LD in this study are 1.35 V and 2 A, respectively. Note that usually a current power supply should be used to drive an LED or LD. Our power supply can function as either a voltage or current source. The IR light is focused on the sample via a lens (LA1805-ML, Thorlabs, NJ, USA) to heat the sample (the laser beam size can be found in Supplementary Information). Additionally, a light blocker (LB2, Thorlabs, NJ, USA) was positioned after the sample to absorb excess infrared light from the LD for safety. An IR thermal camera (C3-X, Teledyne FLIR, OR, USA) was adopted to monitor the surface temperature of the sample (i.e., the cuvette). Thermal cameras usually have high sensitivity and low accuracy^17^. Based on the manufacturer’s manual, this camera has a sensitivity of < 70 mK and an accuracy of ± 3 °C. This means a subtle temperature change on the surface of the sample can be detected via this IR camera, but the absolute value of the measured temperature may not be accurate enough. A high sensitivity is needed for this study because the LD-induced temperature rise is small (~ 3 °C). All the optical components are positioned and locked on two optical breadboards (12″ × 12″ each, MB12, Thorlabs, NJ, USA) and optically shielded by black hardboards (TB4, Thorlabs, NJ, USA) for reducing environmental light (see Fig. 1b).

To be able to heat the sample efficiently using the LD, we selected a cuvette with a very small volume (0.7 mL, CV10Q7F, Thorlabs, NJ, USA). Fluorescein aqueous solution (~ 20 μM) was injected into the cuvette and sealed with a cover. The LED was turned on to excite the sample and the phone camera was used to record videos of the fluorescence signals at a sampling frequency of 30 Hz (frames/s, determined by the phone camera). Then, the LD was turned on to heat the sample. During the heating, we used the phone camera and the IR camera to record the fluorescence signal from the sample and the temperature on the cuvette surface. When heating was done, the LD was turned off but the other components (LED, phone camera, and IR camera) remained on for recording additional time. To measure the temperature sensitivity of the fluorophore (%/°C), the sample cuvette was submerged into a hot water bath (~ 50 °C) for heating for approximately 2 min and then positioned back into the system to measure the fluorescence change during the cooling procedure. The recorded videos were processed and digitized, and the temperature sensitivity was calculated based on the methods discussed in the “Data processing methods” section. All the major components are summarized in Table 1 with detailed information, including rough costs.

**Table 1:** A list of all the major components of the system.

Component name	Model number	Major parameters	Cost
Laser diode	Thorlabs, L1550G1	1550 nm and 1.7 W	~ $350
LED	Thorlabs, LED465E	465 nm and 20 mW	~ $25
Light blocker	Thorlabs, LB2	1–12 µm and 80 W Max Avg	~ $180
Optical breadboard (2x)	Thorlabs, MB12	12″ × 12″ × 1/2″ and 1/4″-20 Taps	~ $344 ($172 each)
Lens tube 1″, (5x)	Thorlabs, SM1L10-P5	1.00″ thread depth	~ $75 ($15 each)
Lens with SM1 threaded mount (4x)	LA1805-ML	Plano-convex lens, 1″ diameter focal length = 30 mm	~ $160 ($40 each)
Post holder (8x)	Thorlabs, PH3-P5	Ø1/2″ post holder and L = 3″	~ $45
Base	Thorlabs, BA1S-P5	1″ × 2.3″ × 3/8″	~ $25
Post (8x)	Thorlabs, TR3-P5	Ø1/2″ post, 8–32 setscrew, 1/4″-20 Tap, L = 3″	~ $25
SM1 square holder retaining rings (4x)	Thorlabs CP33	Threaded 30 mm cage plate, 0.35″ thick, 2 retaining rings, 8–32 Tap	~ $20
Filter 1	Thorlabs, FELH0550	Ø25.0 mm longpass filter, 550 nm	~ $140
Filter 2	Thorlabs, FELH0500	Ø25.0 mm longpass filter, 500 nm	~ $140
Cuvette	Thorlabs CV10Q7F	700 µL microfluorescence cuvette with cap, 10 mm path length	~ $210 (including two cuvette in one page)
Black hardboard	Thorlabs TB4	24″ × 24″ (610 mm × 610 mm) and 3/16″ (4.76 mm) Thick	~ $75 (including 3 sheets)
Structural rail (4x)	Thorlabs XE25L09	25 mm square construction rail, 9″ long, 1/4″-20 taps	~ $70 (~ $17 each)
Power supply (2x)	Digikey KD3005D	Voltage supply: 110–220 VAC Voltage output: 0–30 VDC Current output: 0–5A	~ $180 (2 × at $90 each)
MOSFET (5x)	Goford semiconductor G30N02T	N-channel 20 V 30A (Ta) 40W (Ta)	~ $3 (5 × at $0.57 each)
Thermal camera (can be replaced by other cheaper thermometers)	FLIR C3-X	Handheld IR camera − 20 to 300 °C (− 4 to 572 °F)	~ $590
Uno board	ELEGOO EL-KIT-003	UNO Project Super Starter Kit and UNO R3 Compatible with Arduino IDE	~ $45

### Control system

Although it is not required in the current study, we designed a switching system to control the two power supplies and then the LED and LD (Figs. 1, 2). Such a system will provide the following benefits: (1) it allows the system to be switched on quickly to avoid delay induced by manual switching, (2) it provides the capability to more accurately control the timing among different events, such as synchronizing/controlling the LED, LD, and cameras, which will be necessary for more advanced investigation, and (3) it offers opportunities for students to gain electronics knowledge and learn coding skills.

Figure 2 displays the switching circuit responsible for activating and deactivating the LD and LED. This circuit interfaces with a UNO board (ELEGOO, EL-KIT-003, Amazon, US) via pins twelve (High/Low, + 5/0 V) for the LD and thirteen (High/Low, + 5/0 V) for the LED, enabling the board to control the LD and LED. An essential component in the circuit is the metal oxide semiconductor field-effect transistor (MOSFET), featuring drain (D), sink (S), and gate (G) pins. The MOSFET functions as a logic-level switch, allowing current to flow between D and S. When a voltage higher than a threshold (> 1 V) is applied to the gate (G), the resistance between D and S is so low that the circuit is closed (i.e., connected), and the current can flow through the LED or LD to switch them on. Adjusting the voltage at G via a resistor (PO1) as a voltage divider can control the resistance between D and S, affecting the LD or LED’s power. The voltage at pin twelve or thirteen can be programmed either low (0 V, off) or high (+ 5 V, on) (see the Arduino code in the Supplementary Information for one example). To protect the LED from accidentally overloading, a resistor (R01) can be used to limit the current (but it is not required). It was not used for LD due to the high current (2 A). In the current study, the ground on the UNO board is connected to the ground of the power supply, which is also connected with the negative pin on the power supply and the case pin on the LD, so that the UNO board and the power supply can have a common voltage reference. The UNO board is connected via a USB cable to a computer that contains the code.

### Data processing methods

After videos were acquired by the phone camera, they were transferred to a computer and processed via Matlab (Mathworks, MA, US). A sample script code is attached in the Supplementary Information. Briefly, the video file with an extension of .mov was first read by a function of ‘VideoReader’, which acquired the video properties, such as frame rate and pixel numbers. Users can select a frame range based on their interests. A function of ‘read’ was used to read the frames in the selected frame range. Usually, RGB filters and algorithms are adopted in a phone camera to generate color images. The specific spectrum information and processing methods may not be available. To avoid any potential uncertainties caused by the unknown parameters of the phone camera, we converted the color image into a grayscale image using a Matlab function of ‘rgb2gray’. Thus, each frame provided the brightness of the fluorescence signal. In addition, instead of using an absolute value of the grayscale, we used a percentage change of the fluorescence signal per degree to characterize the temperature sensitivity (see Eq. below). Then, a region of interest (ROI) was selected around the maximum intensity area using a square, a circle, or a percentage of the maximum intensity on the first frame of the selected frame range. Note that in this study a square is adopted because it is much easier to code compared to others. Once ROI was selected, it remained unchanged for all the following frames. The data in the ROI on each frame was spatially averaged and the mean value was used to represent the fluorescence intensity for the time when the frame was acquired. The acquisition time (i.e., the variable of ‘time’) was calculated based on the selected frame range and the frame rate. To further reduce the noise, a moving average filter (see the variable of ‘fil_temp’ and the function of ‘imfilter’) was used to process the data. The sample temperature was directly read from the IR camera images by selecting the ROI on the sample. To evaluate the excitation light leakage from the filters, water samples without fluorophores were also measured using the same procedures. The result can be used as background and subtracted from the fluorescence data if it cannot be ignored. In the current setup, this background is usually ignorable at the non-edge area of the cuvette image because two filters are used.

## Results and discussion

### Circuit test

**Figure 3:**
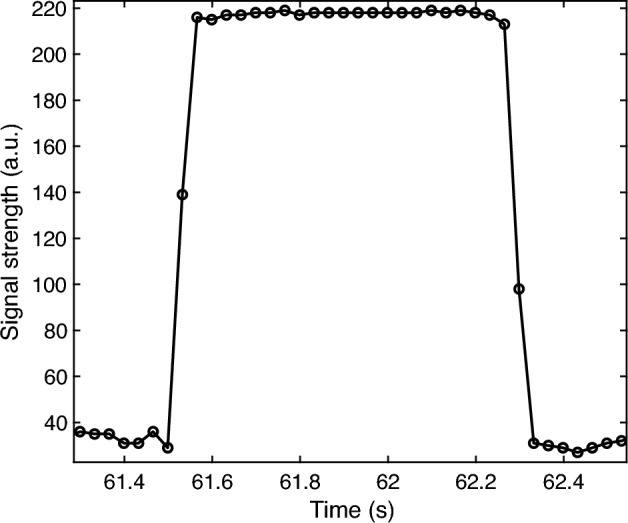
Data of an LED switching test.

To test the switching property of the circuit, the LED was switched on and off. A video was recorded directly and processed using the method described above. The data is shown in Fig. 3. Clearly, the LED switching started at 61.4985 s from a background light level of ~ 30 (arbitrary unit, a.u.) (measured in a regular room environment during daytime) and was completed at 61.5651 s with a light level of ~ 217, which took about 66 ms and was fast enough for most intensity-based applications. Similar results were achieved for the switching off, which started at 62.2651 s and was completed at 62.3312 s.

### Fluorescence temperature sensitivity of the fluorophore

**Figure 4:**
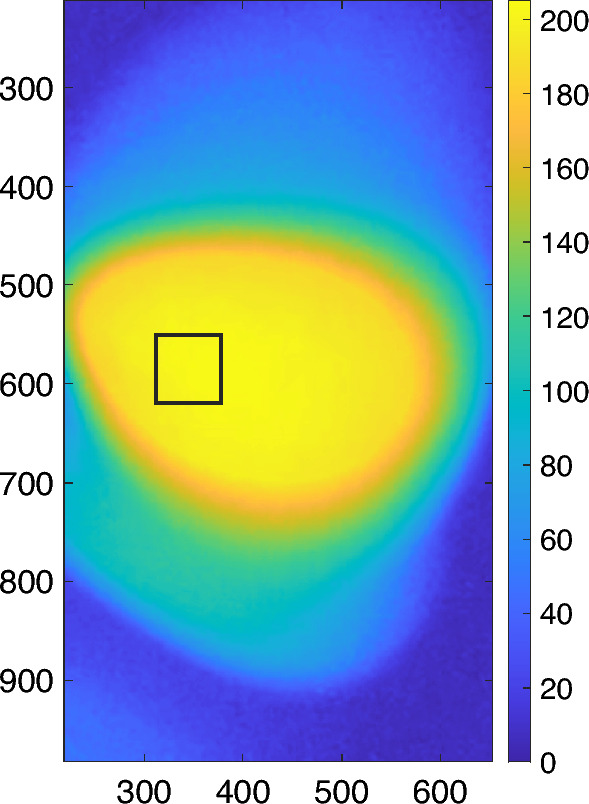
A colored and zoomed-in 2D fluorescence image acquired from the phone camera. The black square shows the ROI.

**Figure 5:**
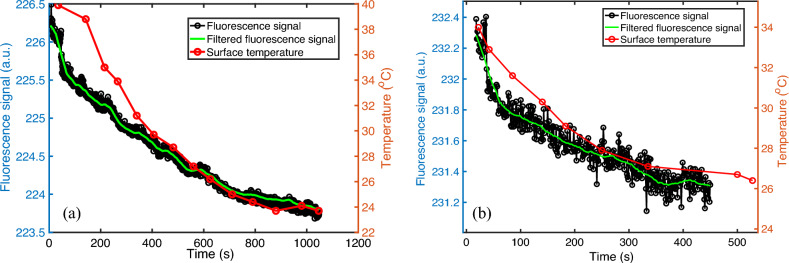
Fluorescence signal (black circles, left axis), moving average filtered data (green line, left axis), and temperature (red circles, right axis) during the sample cooling.

**Figure 6:**
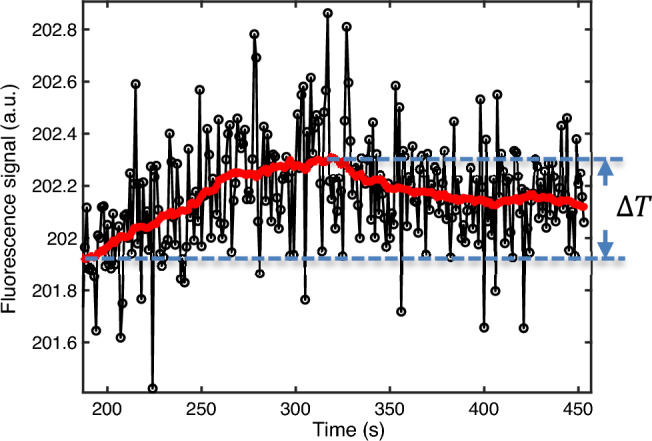
Fluorescence signal (black circles), moving average filtered data (red line), and estimated temperature rise (blue dashed lines) induced by the LD heating.

Figure 4 shows a colored and zoomed-in 2D fluorescence image directly acquired from the phone camera. The black square shows the ROI in which the data were extracted for analysis based on the method described above. Figure 5a,b show two examples of the measured dynamic decays of the fluorescence and temperature during the sample cooling period for calculating the temperature sensitivity of the fluorophore. The left axis is the fluorescence signal, and the right axis represents the measured surface temperature of the cuvette. The black circles and the green line represent the fluorescence signal without and with the moving average filter processing, respectively. The red circles are temperature. An interesting phenomenon can be observed: The initial temperature decay does not match the fluorescence decay, but later they overlap well. This indicates that at the beginning the surface temperature of the cuvette is not the same as the real temperature of the fluorescence sample (i.e., the solution) inside the cuvette. This may be because when the cuvette was just taken out of the water bath the cuvette surface was hotter than the sample solution inside the cuvette. However, after the cuvette was positioned on the measurement system, its surface temperature was reduced faster than the sample temperature, and gradually both reached an equilibrium. Other possible error sources may come from measurement variations due to the IR camera's high sensitivity and low accuracy, and the experimental (such as operational and/or environmental) uncertainties. Another phenomenon can be observed by comparing Fig. 5a with Fig. 5b: Higher temperature change ($\Delta T$: 16.2 vs 9.2 °C) can induce larger fluorescence change ($\Delta F$: 2.404 vs 0.942 a.u.), and therefore a less noisy curve in Fig. 5a than in Fig. 5b. This phenomenon will be further seen in Fig. 6, which illustrates using the LD to heat the sample.

The fluorescence temperature sensitivity ${S}_{FT}$ can be calculated via the following Eq.^17^,

$$S_{FT} = \frac{{\Delta F/\left( {\left. {F_{0} - F_{BG} } \right)} \right.}}{\Delta T} \times 100\%$$

In which $\Delta F$ is the fluorescence signal difference between any two selected time points of ${t}_{1}$ and ${t}_{0}$ (i.e., ${F}_{1}\left({t}_{1}\right)$–${F}_{0}\left({t}_{0}\right)$), $\Delta T$ is the corresponding temperature difference (i.e., ${T}_{1}\left({t}_{1}\right)$–${T}_{0}\left({t}_{0}\right)$), ${F}_{0}$ is the fluorescence signal at ${t}_{0}$ (i.e., ${F}_{0}\left({t}_{0}\right)$), and ${F}_{BG}$ is the background data acquired from the water sample (this is ignorable in our study after using two filters). To avoid the temperature error at the beginning, the fluorescence temperature sensitivity was calculated only based on those data where the two curves overlapped reasonably in Fig. 5. Thus, Fig. 5a provides a $S_{FT} = 0.065\%$/°C (i.e., [(224.682 − 223.808)/(223.808)/(29.7 − 23.7)] * 100%/°C) and Fig. 5b indicates a $S_{FT} = 0.053\%$/°C (i.e., [(231.589 − 231.309)/(231.309)/(29.1 − 26.8)] * 100%/°C). The mean and standard deviation of ${S}_{FT}$ were finalized as 0.059 and 0.0085 (%/°C), respectively. This sensitivity is much smaller than the 3.5%/°C reported in the literature for the same fluorophore when using a 532 nm laser as the excitation light (a 465 nm light source was used in current study)^1^. This phenomenon of temperature sensitivity dependence on the excitation light wavelength has been reported in the literature^1^.

Note that Fig. 5a,b are two different measurements corresponding to two times of heating via the water bath (but using the same sample). Therefore, the absolute value of the signal should not be compared because (1) the sample might be positioned back slightly differently from the 1st time on the holder and (2) the selected area for data processing in the code may be different. In this study, we do not compare any absolute values of the fluorescence signal.

### Estimation of temperature rise induced by LD heating

Figure 6 shows the measured fluorescence signal (black circles) and the moving average filtered data (red line) when the LD was turned on at ~ 188 s to heat the sample and off at ~ 319 s. The non-filtered data (black circles) are noisy because the LD-induced temperature rise is only ~ 3 °C (measured from the IR camera at the cuvette surface), which is much lower than the temperature rise induced by the water bath discussed in Fig. 5. After filtering, the data quality is significantly improved. The heating and cooling trends can be clearly recognized. Based on the filtered data and the average temperature sensitivity of the fluorophore measured above, the temperature change $\Delta T$ induced by the LD can be estimated as 3.2 °C (i.e., (202.313 − 201.931)/201.931 * 100 (%)/0.059 (%/°C)), which is slightly higher than the 3 °C measured by the IR camera (a thermal image of the heated sample by the LD can be found Fig. S1 in Supplementary Information).

### LD overheating issue

Due to a large current being applied (2 A in the current study), the LD itself can be easily overheated, especially when the operation time is > 5–10 min. The case temperature of the LD can reach as high as 65 °C (measured using the IR thermal camera), which is significantly higher than the manufacturer-specified case temperature range (10–30 °C). This issue leads to the illumination intensity from the LD dimming gradually. To avoid this problem, the temperature of the LD should be cooled and controlled, which can be achieved using an LD mount with a temperature controller. However, these items are usually expensive (> $1000). Fortunately, in this study, LD-induced heating as high as ~ 3 °C can be achieved within a few minutes by using a small volume cuvette. By doing this, combined with the “Data processing methods” described above, the fluorescence change can be observed clearly enough to estimate the temperature rise before the LD dims significantly. On the other hand, the current applied to the LED in this study is only 9 mA. Its case temperature can stabilize around 28 °C after being switched on. This is within its operating temperature range (− 30 to 85 °C). Therefore, the LED can provide a stable output power.

### Photobleaching and baseline drifting/stability

**Figure 7:**
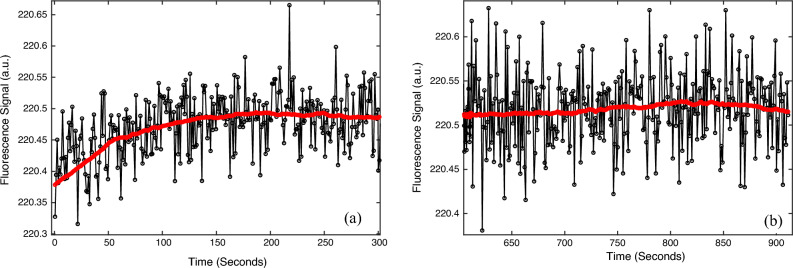
Fluorescence signal (black circles) and moving average filtered data (red line) at room temperature (without heating): (**a**) baseline drifting example, and (**b**) stabilized baseline example.

Photobleaching frequently happens in fluorescence measurements, which is usually represented as the reduction of emission strength due to long-time excitation light illumination. To investigate this effect, the sample was continuously excited for about 48 min and the fluorescence was acquired. The data showed that no continuous fluorescence reduction could be identified (Fig. S2). Therefore, photobleaching should not be an issue for the adopted fluorophore and experimental conditions in the current study. However, sometimes it was possible to observe small baseline drifting (Fig. 7a, 0.05% = (220.490 – 220.379)/220.379 * 100). The reason is not clear, but it is possibly due to the LED or camera response thermal stabilization. Fortunately, the system can be stabilized eventually (Figs. 7b and S2). It may be helpful to check the baseline stability before acquiring data to avoid possible baseline drifting. However, if this drifting happens during the experiment, it may lead to about 0.85 °C (= 0.05%/0.059%/°C) overestimation of the temperature in the current setup.

### Further discussion

We realized that a homemade system has been adopted for measuring the fluorescence properties of a temperature-sensitive nanoprobe for thermal ablation applications^19^. Briefly, our work mainly aims to make a cost-effective system for quantifying fluorophore temperature sensitivity and temperature change. Therefore, we developed a phone camera-based approach, including the data processing method. It is commonly seen in the literature that a cooled camera with commercial software is used for fluorescence detection, which usually is much more expensive, bulkier and less widely available compared with a phone-based camera. In addition, compared with a heat gun, an IR LD can heat the sample in a well-controlled way by adjusting the supplied current, especially when the temperature increase is small, and/or highly localized heating is required.

It is worth pointing out that phone cameras usually have filters (red, green, and blue) in front of their sensors and some algorithms for converting data. The filter spectra and algorithms may not be disclosed by the manufacturers. Using the relative change of fluorescence signal and converting color images to grayscale may be helpful to avoid or minimize their potential effects among different phone cameras.

It has been reported that, for the same fluorophore, a linear relationship exists between the fluorescence signal and the concentration when the concentration is lower than ~ 3.5 µM^1^. After that, the slope of the line is gradually reduced, which is commonly seen in the literature and may be mainly caused by the inner filter effect and self-quenching^1^. In this study, the small sample volume can help to minimize the inner filter effect, but the self-quenching cannot be excluded (because our sample concentration is 20 µM).

Allan deviation (or variance) is a good method to analyze the noise and guide the fitting parameter (such as the filter size)^20^. As an example, we used the data in Fig. 7a with a sample rate of 1 Hz to analyze the noise (Fig. S3). The result shows that when the sample time τ = 32 the deviation reaches a minimum value, which indicates the best temporal filter length.

## Conclusions

In summary, a cost-effective system was designed and tested for measuring fluorophore temperature sensitivity and temperature change. An LED (465 nm) and an LD (1550 nm) were used to excite fluorescence and induce sample temperature increase. A phone camera was used to record the fluorescence change and a data processing method was developed to convert the video into digital data and further process the data. To efficiently increase the sample temperature, a cuvette with a small volume (0.7 mL) was proposed and tested. The estimated temperature sensitivity of the adopted fluorophore (fluorescein 27) is about 0.059%/°C, which is much smaller than the 3.5%/°C reported in the literature. This is mainly attributed to the difference in the adopted excitation light wavelength and detection wavelength. In addition, without cooling the LD, the sample temperature can be increased by about 3.2 °C in a few minutes, which can be clearly observed from the fluorescence rising. Therefore, the system and methods described in this study can function as a valuable guide for inexperienced students, instructors, and researchers to get started in this field and further develop their skills, methods, and techniques for different applications.

### Supplementary Information

Supplementary Information.

## Data Availability

All data generated or analysed during this study are included in this published article.
