# Supplementary material for: A cost-effective approach to measurements of fluorophore temperature sensitivity and temperature change with reasonable accuracy
Source: Sci Rep. 2024 Mar 21;14:6823. doi: 10.1038/s41598-024-57387-2 (PMC10957993; doi:10.1038/s41598-024-57387-2)
Supplement: Supplementary file 1 — Supplementary Information. [file 41598_2024_57387_MOESM1_ESM.docx]

**Supplementary Information**

**A cost-effective approach to measurements of fluorophore temperature sensitivity and temperature change with reasonable accuracy**

Matthew Cai ^+^, Alexander Sun ^+^, Andrea Yan, Zachary Ding, Melvin Zunyao Jiang, Charissa Wang, Baohong Yuan *

SRCP, RCLabX LLC, Southlake, TX, 76092

^+^ These authors contributed equally to this work.

* Corresponding author: [infosrcp2022@gmail.com](mailto:infosrcp2022@gmail.com)

The following codes are just examples, and the specific values for the parameters may not be the same as the ones used in the data acquisition given in the figures.

**S1. Arduino code:** The following code was used as an example to show how to switch on and off (i.e., modulate) the light sources via the circuit. The parameter values can be adjusted. Lines 1-7 set up the code and define each pin, telling the system the location of each pin and defining whether the pin is inputting information or running the output of the code. Lines 2 and 3 define the LED and LD pins, telling what number the pins are, and lines 4 and 5 define whether the pin intakes information to supply to the code or vice versa. Lines 8-13 give integers to define the configurations of the variables of the code. In line 14, the code begins being looped. Line 15 processes the seconds configuration from above to actual seconds instead of milliseconds as the blink interval of the LED. The following ‘if’ clause resets the LED and flashes it. In line 22, the LD interval is divided by the LED interval to achieve an LD interval consistent with the inputted configurations and applies the delay to the LD flash. Lines 24 through 28 reset the LD and flash it according to the above division. In line 31, the configuration of the LED interval is inputted as a delay to the flash of the LED. At the end of the code, the loop is terminated.

1. void setup() {
2. #define LED_PIN 13 //tells the system that LED is digital Pin 13
3. #define LD_PIN 12 //tells the system that LD is digital Pin 12
4. pinMode(13, OUTPUT); //set digital pin 13 as output
5. pinMode(12, OUTPUT); //set digital pin 12 as output
6. }
7. int seconds =10; //amount of seconds you want to have the system active
8. int laserinterval =500; //how long you want the LD interval to be(ms)
9. int ledinterval =200; //how long you want the LED interval to be(ms)
10. int c =0; //to count iterations for the LD
11. int i =0; //to count iterations for the entire program
12. void loop(){
13. while(i <(seconds *1000)/ledinterval){
14. if(digitalRead(LED_PIN) ==HIGH){
15. digitalWrite(LED_PIN, LOW);
16. }
17. else{
18. digitalWrite(LED_PIN, HIGH);
19. }
20. if(c >= laserinterval /ledinterval){
21. c =0;
22. if(digitalRead(LD_PIN) ==LOW){
23. digitalWrite(LD_PIN, HIGH);
24. }
25. else{
26. digitalWrite(LD_PIN, LOW);
27. }
28. }
29. delay (ledinterval);
30. i +=1;
31. c +=1;
32. }
33. digitalWrite(LED_PIN, LOW);
34. digitalWrite(LD_PIN, LOW);
35. }

**S2. Matlab code:** The following code was used as an example to show how to convert a video file into digital data and how to process the data via two digital filters to be able to visualize the fluorescence signal change caused by temperature change. The parameter values can be adjusted.

1. clear all
2. close all
3. perc=1; ext=30; frame_stp=30; fil_size=20; fil_size_temp=20; % these numbers are adjustable
4. fil=ones(fil_size,fil_size)/(fil_size.*fil_size); % create the normalized filter matrix in spatial domain
5. fil_temp=ones(1, fil_size_temp)/(fil_size_temp); % create the normalized filter matrix in time domain
6. vidObj = VideoReader('IMG_1.mov'); % read the video properties
7. frame_range_time=[0.1 17.5]*60; % specify the time range, seconds*60=minutes
8. frames_range=[floor(frame_range_time(1)./(1./vidObj.FrameRate)) floor(frame_range_time(2)./(1./vidObj.FrameRate))]; % convert the time range into frame range
9. frames = read(vidObj,frames_range); % read the data from the selected frames
10. n=size(frames,4);
11. frame_total=[1:frame_stp:n]; % choosing the frames via the frame step size
12. ast=double(rgb2gray(frames(:,:,:,1))); % convert the RGB data into grayscale data
13. [xx, yy]=find(ast==floor(max(max(ast))*perc)); % find the parameters for ROI
14. figure; imagesc(ast); axis image; colorbar; % plot the 1^st^ frame of the selected frames
15. hold on; plot(yy, xx, 'ks'); % add the parameters on the 1^st^ frame image
16. for i=1:length(frame_total) % processing the selected frames
17. newframe1(:,:,i)=double(rgb2gray(frames(:,:,:,frame_total(i)))); % convert the RGB data into grayscale data
18. A=squeeze(newframe1(:,:,i));
19. newframe=imfilter(A,fil,'replicate'); % apply the spatial filter
20. int_time_1(i)=mean(mean(newframe([xx(1)-ext:xx(1)+ext], [yy(1)-ext:yy(1)+ext]))); % calculate the mean of the data within the ROI
21. end
22. time=(1./vidObj.FrameRate).*(frame_total+min(frames_range)-1); % acquisition time
23. int_time_fil=imfilter(int_time_1,fil_temp,'replicate'); % apply the temporal filter
24. [avar, tau]=allanvar(int_time_1, 'octave', 1/(time(2)-time(1)));
25. figure; loglog(tau, sqrt(avar), 'linewidth', 3)
26. xlabel('\tau'); ylabel('Allan Deviation')
27. set(gca, 'box', 'on','fontsize',24,'Linewidth',3);
28. figure; plot(time, int_time_1, '-ok', 'Linewidth',3, 'Markersize', 10); % plot the data without temporal filter
29. hold on; plot(time, int_time_fil, '-g','Linewidth',3, 'Markersize', 10); % plot the data with the temporal filter
30. xlabel('Time (s)','Fontsize', 24);
31. ylabel('Fluorescence signal(a.u.)','Fontsize', 24)
32. set(gca, 'YScale', 'linear', 'XScale', 'linear', 'box', 'on','fontsize',24,'Linewidth',3);
33. figure; imagesc(ast); axis image; colorbar; % plot the 1^st^ frame of the selected frames
34. hold on; plot([yy(1)-ext:yy(1)+ext], [xx(1)-ext:xx(1)+ext], 'bo') % add the ROI on the image

**S3. The laser diode beam and heating results**

As described in the system introduction, a lens (LA1805-ML, Thorlabs, NJ, USA) was used to focus the laser diode (LD) beam on the sample. The laser beam shows the shape of a stripe on the sample surface (detected by a VIS/IR detector card, VRC2, Thorlabs, because IR light is invisible to human eyes). The rough size of the laser beam is about 3x20 mm. Because the sample volume is very small (<0.7 ml) and heating time is at a level of minutes, thermal energy can sufficiently diffuse to the entire volume. Thus, the heated volume is much bigger than the laser beam size, which can be seen from a thermal image (see Fig.S1). Although we do not expect that the sample was heated up completely homogeneously, as long as the setup is fixed during the experiment, the data should reflect the percentage change of the fluorescence per degree of the heated-up volume (which was fixed during the experiment). Of course, if available, a beam collimator and expander may be able to illuminate the sample more uniformly, but it may increase the cost.

**
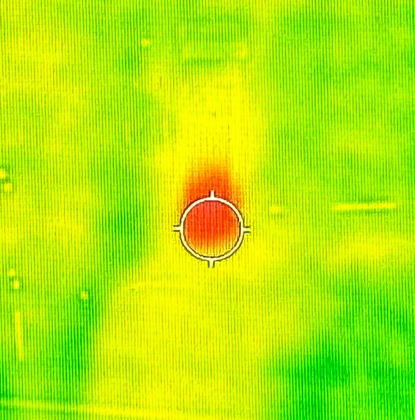
**

Fig.S1: A typical thermal image of the sample after being illuminated by the LD as described in Fig.6. The temperature increase was 3 degrees.

**S4. Photobleaching**

Fig.S2 displays the acquired background fluorescence signal over a long period (48 minutes). During this period, the LED was always turned on. To avoid acquiring a large-sized video, which would lead to difficulty for Matlab to process, two videos were acquired and shown in the figure.

The photobleaching-caused signal continuous reduction cannot be identified based on the data in either group.

Fig.S2: Fluorescence signal acquired over a long period of 48 minutes.

**S5. Allan deviation**

Allan deviation (or variance) is a good method to analyze the noise and guide the fitting parameter (such as the filter size). As an example, Fig.S3 shows a log-log plot of the Allan deviation (i.e. the square root of Allan variance) vs sample time τ (seconds, at 1 frame/second). Note that the camera’s actual acquisition frame rate is ~30 Hz (i.e. 0.0334 s per frame), which is too high for our application and makes the data processing difficult (limited by the Matlab). In our processing (see the variable of “frame_stp” in the Matlab code), we reduced the frame rate down to 1 Hz, which meant only one frame was taken for processing every second. Thus, the sampling frequency was 1 Hz for calculating the Allan deviation (see the function of “allanvar”). The signal data were taken from Fig.7(a). The result shows a minimum value of τ=32, which indicates the best temporal filter length. In practice, selecting a number equal to or around this number should be a good choice.

Fig.S3: Allan deviation vs sample time τ (seconds, at 1 frame/second)
